# Clinical Impact of Speed Variability to Identify Ultramarathon Runners at Risk for Acute Kidney Injury

**DOI:** 10.1371/journal.pone.0133146

**Published:** 2015-07-15

**Authors:** Sen-Kuang Hou, Yu-Hui Chiu, Yi-Fang Tsai, Ling-Chen Tai, Peter C. Hou, Chorng-Kuang How, Chen-Chang Yang, Wei-Fong Kao

**Affiliations:** 1 Department of Emergency Medicine, Taipei Medical University Hospital, Taipei, Taiwan; 2 Institute of Environmental and Occupational Health Sciences, National Yang-Ming University, Taipei, Taiwan; 3 Department of Emergency Medicine, Brigham and Women’s Hospital, Boston, Massachusetts, United States of America; 4 Department of Emergency Medicine, Mackay Memorial Hospital, Taipei, Taiwan; 5 Department of General Surgery, Taipei Veterans General Hospital, Taipei, Taiwan; 6 Institute of Clinical Medicine, National Yang-Ming University, Taipei, Taiwan; 7 Harvard Medical School, Boston, Massachusetts, United States of America; 8 Department of Emergency Medicine, Taipei Veterans General Hospital, Taipei, Taiwan; School of Public Health of University of São Paulo, BRAZIL

## Abstract

**Background:**

Ultramarathon is a high endurance exercise associated with a wide range of exercise-related problems, such as acute kidney injury (AKI). Early recognition of individuals at risk of AKI during ultramarathon event is critical for implementing preventative strategies.

**Objectives:**

To investigate the impact of speed variability to identify the exercise-related acute kidney injury anticipatively in ultramarathon event.

**Methods:**

This is a prospective, observational study using data from a 100 km ultramarathon in Taipei, Taiwan. The distance of entire ultramarathon race was divided into 10 splits. The mean and variability of speed, which was determined by the coefficient of variation (CV) in each 10 km-split (25 laps of 400 m oval track) were calculated for enrolled runners. Baseline characteristics and biochemical data were collected completely 1 week before, immediately post-race, and one day after race. The main outcome was the development of AKI, defined as Stage II or III according to the Acute Kidney Injury Network (AKIN) criteria. Multivariate analysis was performed to determine the independent association between variables and AKI development.

**Results:**

26 ultramarathon runners were analyzed in the study. The overall incidence of AKI (in all Stages) was 84.6% (22 in 26 runners). Among these 22 runners, 18 runners were determined as Stage I, 4 runners (15.4%) were determined as Stage II, and none was in Stage III. The covariates of BMI (25.22 ± 2.02 vs. 22.55 ± 1.96, *p* = 0.02), uric acid (6.88 ± 1.47 vs. 5.62 ± 0.86, *p* = 0.024), and CV of speed in specific 10-km splits (from secondary 10 km-split (10^th^ – 20^th^ km-split) to 60^th^ – 70^th^ km-split) were significantly different between runners with or without AKI (Stage II) in univariate analysis and showed discrimination ability in ROC curve. In the following multivariate analysis, only CV of speed in 40^th^ – 50^th^ km-split continued to show a significant association to the development of AKI (Stage II) (*p* = 0.032).

**Conclusions:**

The development of exercise-related AKI was not infrequent in the ultramarathon runners. Because not all runners can routinely receive laboratory studies after race, variability of running speed (CV of speed) may offer a timely and efficient tool to identify AKI early during the competition, and used as a surrogate screening tool, at-risk runners can be identified and enrolled into prevention trials, such as adequate fluid management and avoidance of further NSAID use.

## Introduction

Ultramarathon, a foot race longer than the standard 42.2 kilometer (km) marathon distance, has become increasingly popular in recent decades throughout the world [1]. During this endurance event, athletes provoke many physiological responses and metabolic adaptations to finish the competition [2]. However, higher level of the physiological demands may induce a wide range of exercise-related injuries, such as electrolyte imbalance [3,4], exertional heat stroke [5,6], rhabdomyolysis, acute kidney injury [7–9], and even cardiac arrest [10,11]. With the growing numbers of participants, early recognition of runners at risk for the development of exercise-related injuries is paramount.

In the literature, several studies reported that 40–80% of athletes developed acute kidney injury (AKI) after strenuous exercise [12–14]. The pathophysiology of exercise-related AKI is multi-factorial [15] and may be the combined effect of dehydration, hot environment [16,17], nonsteroid anti-inflammatory drugs use [18,19], hyperuricemia [9,15], and rhabdomyolysis [7–9]. During ultramarathon, early recognition and management of runners at risk of impending kidney injury/failure are critical to prevent serious complications. Although the diagnosis of AKI can be made by checking the serum creatinine level before and after the race, it may not be feasible to identify individuals at risk of the development of AKI early during the ultramarathon event.

Although the running speed of ultramarathon might be influenced by several factors such as altitude changes, temperature, humidity, and fatigue, runners strategize to maintain appropriate speed in response to physical condition to avoid falling into over-fatigued [20–22]. Some studies reported that the variability of speed may affect the performance and metabolic demand in different-distance running [23–26]; however, there is no published data evaluating the relationship between variability of speed and exercise-related injuries, such as AKI, during ultramarathon running. The aim of the study is to investigate the association of speed variability to identify exercise-related acute kidney injury during an ultramarathon event.

## Materials and Methods

### Study design and population

Experienced ultramarathon runners participating in the 2011 Flexpower Cup National 100 km Ultra-Marathon in Soochow University, Taipei, Taiwan, were enrolled in this study. Registered participants were contacted by phone to explain the study protocol, its objectives, and to determine each participant’s willingness to volunteer for the study. Runners were excluded if they had past histories of heart disease, renal dysfunction, seizure, or syncope of unknown origin.

All runners ran over a flat course consisting of a 400 m oval track. During the race, runners wore a racing bib with a timing chip to record each lap time automatically at every 400 m intervals by RFID (Radio-frequency identification) timing system. They were permitted to rest, micturate, and consume water or food. All runners completed a pre-race questionnaire for demographic data, medical information (such as NSAID used within one week), and training history before this competition.

### Ethics Statement

Institutional Review Board approval (VGHIRB No: 2011-01-060IC) was obtained from the Ethics Committee of Taipei Veterans General Hospital. All subjects were contacted by phone using information provided by the Chinese Taipei Association of Ultra Runners and then provided written consent to participate in the study.

### Data collection

Lap times were obtained to measure the running speed (m/s) every 400 meters. After dividing the distance of entire ultramarathon race (100 km) into 10 splits, the mean and standard deviation (SD) of running speed in each 10 km-split (25 laps of 400 m oval track) were calculated for all runners. The variability of speed was determined by using the coefficient of variation (CV), which was defined as the ratio of the standard deviation to the mean of lap speeds [23,26,27].

Venous blood (20ml) was drawn antiseptically by a 20-gauge intravenous catheter 1 week before and immediately post-race to examine biochemical data, including blood urea nitrogen (BUN), creatinine (Cr), creatine kinase (CK), lactate dehydrogenase (LDH), myoglobin, uric acid (UA), electrolytes, D-dimer, Procalcitonin, and liver function tests. BUN and Cr were examined again one day after race to assess the recovery of renal function. All specimens were refrigerated and transported to the laboratory within 4 hours of sampling. Plasma samples were assayed on the Siemens Dimension RXL Max Integrated Chemistry System using reagents supplied by the manufacturer. Body weight (BW) change (the difference between before and 4 hours after the start) was also recorded to monitor the dehydrated status.

### Definition of acute kidney injury

Acute kidney injury (AKI) was defined according to AKIN criteria published by Acute Kidney Injury Network [28]. AKI was classified as Stage I (a percentage increase in post-race serum Cr 1.5 to 2-fold from pre-race level, or an absolute increase in serum Cr ≥ 0.3mg/dl), Stage II (a percentage increase in post-race serum Cr 2 to 3-fold from pre-race level), and Stage III (a percentage increase in post-race serum Cr more than 3-fold from pre-race level, or serum Cr ≥ 4mg/dl with an acute increase in serum Cr ≥ 0.5mg/dl, or on renal replacement therapy), respectively. In this study, we used AKI in Stage II or III as the main outcome because Stage I was usually defined to be at-risk of acute kidney injury only.

### Statistical analysis

The baseline characteristics were presented as percentages for categorical variables and mean ± standard deviation (SD) for continuous variables. Paired T test was used to compare differences between pre-race and immediate post the ultramarathon event. Chi-squared test (or the Fisher exact test when appropriate) was applied for categorical data. Baseline characteristics and CV of speed in specific 10 km-splits with significant impact in the initial univariate analysis were retained into further multivariate analysis to examine the independent effects to identify the development of AKI (Stage II or III). To compare the predictive power, we created probability estimate through conducting the multiple logistic regression analysis. Then we apply the predicted probability as test variables of ROC curve to measure the discrimination ability of individualized CV value.

A two-sided p value of 0.05 or less was considered to be significant. All statistical analyses were performed using SPSS software (version 18.0; SPSS Inc., Chicago, IL)

## Results

The ultramarathon began at 7 AM and ended at 9 PM on October 10, 2011. The temperature during this competition was between 24.9°C (7–8 AM) and 28.7°C (5–6 PM), the relative humidity was between 66% (10–11 AM) and 87% (8–9 AM), and the wind speed ranged from 0 m/s (7–8 AM) to 6.5 m/s (11 AM—1 PM).

Total 28 runners (27 male and 1 female) were enrolled in this study. Two subjects unable to complete the 100 km ultramarathon within the predetermined time limit of 14 hours were excluded for further analysis. The median age of all study subjects was 47 years (range from 22 to 60 years old), and ultramarathon experience of these runners was 5.5 ± 2.6 years in average. Before competition, these runners had different training protocol from less than 40 km to more than 100 km per week.

The overall incidence of AKI in all stages was 84.6% (22 in 26 runners); and among them, 4 runners (15.4%) were determined as Stage II of AKI and none in Stage III. Demographics and biochemical data were summarized throughout this highly endurance competition (Table 1). Similar to previous reports [2], there was a statistically significant difference in body weight (BW) and majority of biochemical data between pre-race and immediate post-race exam. Although 3 runners (11.5%) developed asymptomatic exercise-associated hyponatremia (Na ≤ 135 mmol/L), no significant difference was found in serum Na (139.73 ± 1.08 vs. 139.92 ± 3.61, *p* = 0.79) between pre-race and immediately post-race.

**Table 1:** Demographics and biochemical data at pre-race and immediate post-race of all 100 km ultramarathon runners (*n* = 26).

	Pre-race	Immediate Post-race	*P* value
Age (yrs)	49.62 ± 9.02		
Gender (male/female)	25/1		
Ultramarathon experience (yrs)	5.52 ± 2.67		
Body weight (kg)	64.94 ± 9.46	63.31 ±9.15	<0.001
BUN (mmol/L)	15.23 ± 2.89	24.04 ± 5.49	<0.001
Creatinine (mmol/L)	0.94 ± 0.11	1.56 ± 0.39	<0.001
Na (mmol/L)	139.73 ± 1.08	139.92 ± 3.61	0.786
K (mmol/L)	3.99 ± 0.26	4.42 ± 0.39	<0.001
Glu (mg/dL)	93.15 ±13.75	105.46 ±26.29	0.023
AST (U/L)	27.62 ± 7.84	151.12 ± 182.28	0.002
Myoglobin (μg/L)	48.9 ± 20.63	4462.46 ± 3391.87	<0.001
CK (U/L)	166.73 ± 96.91	4274.81 ± 5903.85	0.002
D-Dimer (mcg/mL)	0.29 ± 0.36	0.51 ± 0.51	0.005
Osmolarity (mosm/kgH2O)	290.08 ± 8.83	300.88 ± 7.25	<0.001
Procalcitonin (μg/L)	0.086 ± 0.14	0.44 ± 0.32	<0.001
Uric Acid (mg/dL)	5.82 ± 1.05	7.12 ± 1.30	<0.001

Continuous data presented as mean ± SD.

For the purpose to evaluate the association between pre-race biochemical data and the development of AKI, we compared these variables in runners who did or did not develop AKI (Stage II) in Table 2. Although these biochemical variables changed significantly after race, the majority of the pre-race data didn’t show significant association to AKI (Stage II) development except uric acid (6.88 ± 1.47 vs. 5.62 ± 0.86, *p* = 0.024). Baseline characteristics, including BW change in first 4 hours, pre-race BMI, marathon experience, and weekly training distance were all examined at the same time, and only pre-race BMI was significantly higher in runners developing AKI (Stage II) (25.22 ± 2.02 vs. 22.55 ± 1.96, *p* = 0.02). Other risk factors associated with AKI development, such as NSAID used within one week, were also evaluated by pre-race questionnaires and all runners denied any special medical conditions except one runner in each groups took NSAID.

**Table 2:** Baseline characteristics and pre-race biochemical data were analyzed by independent T-test for the comparison between runners with or without AKI (Stage II) development.

	Normal (*n* = 22)	AKI_Stage II (*n* = 4)	*P* value
Age (yrs)	46.45 ± 9.60	49.50 ± 4.66	0.545
Ultramarathon experience (yrs)	5.67 ± 3.65	4.63 ± 1.49	0.585
Training protocol (/week)			0.331
< 40 km	3 (14.3%)	1 (25%)	
40–100 km	13 (61.9%)	1 (25%)	
> 100 km	5 (23.8%)	2 (50%)	
Uric Acid (mg/dL)	5.62 ± 0.86	6.88 ± 1.47	0.024
BW_change % (4 hrs)	2.16 ± 1.33	1.75 ± 0.76	0.560
BMI	22.55 ± 1.96	25.22 ± 2.02	0.020
BUN (mmol/L)	14.95 ± 2.72	16.75 ± 3.78	0.261
Creatinine (mmol/L)	0.94 ± 0.12	0.95 ± 0.07	0.890
Na (mmol/L)	139.73 ± 1.12	139.75 ± 0.96	0.970
K (mmol/L)	3.98 ± 0.25	4.03 ± 0.34	0.767
Glu (mg/dL)	93.91 ± 14.78	89.0 ± 4.32	0.522
AST (U/L)	27.64 ± 8.34	27.50 ± 5.07	0.975
Myoglobin (μg/L)	49.57 ± 22.41	45.40 ± 6.13	0.720
CK (U/L)	164.14 ± 94.12	181.00 ± 126.24	0.756
D-Dimer (mcg/mL)	0.30 ± 0.28	0.25 ± 0.21	0.789
Osmolality (mosm/kgH2O)	290.24 ± 9.62	289.25 ± 2.36	0.842
Procalcitonin (μg/L)	0.10 ± 0.15	0.03 ± 0.01	0.390

Continuous data presented as mean ± SD.

Definition of abbreviations: BW = Body Weight; BMI = Body mass index.

**Fig 1:**
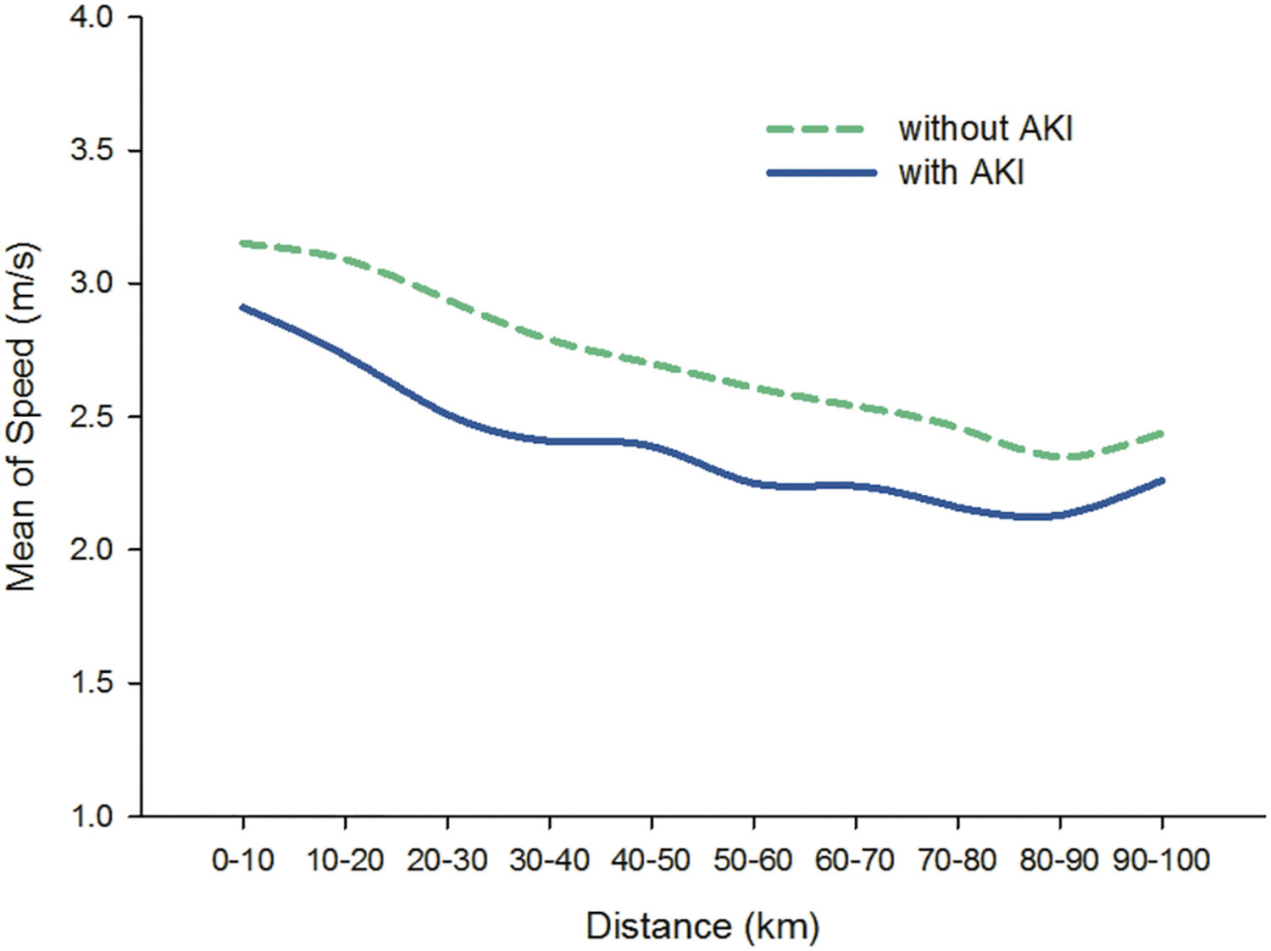
Mean speed of runners in each 10 km-split between runners with or without AKI (Stage II) development. There was no significant difference of mean speed between each group.

During this 100 km ultramarathon race, the average time to complete the 100 km course was 670 ± 85 minutes, with the fastest record of 487 minutes and the slowest record of 827 minutes. The mean running speed and CV in each 10 km-split is shown in Table 3. In our study, mean speed of runners with or without AKI (Stage II) development both decreased gradually following the progress of running distance. The highest mean speed (2.91 ± 0.21 vs. 3.15 ± 0.34 m/s) was detected in the initial 10 km-split (0 – 10^th^ km) and the lowest mean speed (2.13 ± 0.26 vs. 2.35 ± 0.33 m/s) showed in the penultimate 10 km-split (80^th^– 90^th^ km). Due to the sprint before the end, the mean speed in the latest 10 km-split (90th– 100th km) was slightly higher than the 80^th^– 90^th^ km-split in both groups (Fig 1).

**Table 3:** Mean and coefficient of variation (CV) of speed in each 10 km-split between runners with or without AKI (Stage II) development.

Each 10 km-split	Speed, m/s			CV of speed, %		
	Normal (*n* = 22)	AKI Stage II (*n* = 4)	*P* value	Normal (*n* = 22)	AKI Stage II (*n* = 4)	*P* value
0 – 10^th^ km	3.15 ± 0.34	2.91 ± 0.21	0.191	2.63 ± 1.03	3.73 ± 1.31	0.070
10^th^– 20^th^ km	3.09 ± 0.33	2.73 ± 0.24	0.046	4.34 ± 2.27	7.91 ± 2.40	0.009
20^th^– 30^th^ km	2.94 ± 0.38	2.51 ± 0.29	0.041	5.87 ± 3.86	10.74 ± 2.39	0.024
30^th^– 40^th^ km	2.79 ± 0.38	2.41 ± 0.24	0.069	7.24 ± 4.04	13.27 ± 4.18	0.012
40^th^– 50^th^ km	2.70 ± 0.37	2.39 ± 0.21	0.123	8.27 ± 3.92	15.26 ± 2.90	0.003
50^th^– 60^th^ km	2.61 ± 0.34	2.25 ± 0.12	0.045	8.97 ± 4.53	14.47 ± 3.63	0.032
60^th^– 70^th^ km	2.54 ± 0.28	2.24 ± 0.12	0.048	8.46 ± 4.07	13.83 ± 2.87	0.019
70^th^– 80^th^ km	2.46 ± 0.35	2.16 ± 0.22	0.113	8.64 ± 3.99	12.91 ± 6.11	0.081
80^th^– 90^th^ km	2.35 ± 0.33	2.13 ± 0.26	0.209	9.54 ± 4.60	14.07 ± 5.20	0.088
90^th^– 100^th^ km	2.44 ± 0.39	2.26 ± 0.35	0.400	9.80 ± 4.69	14.78 ± 8.77	0.101

**Fig 2:**
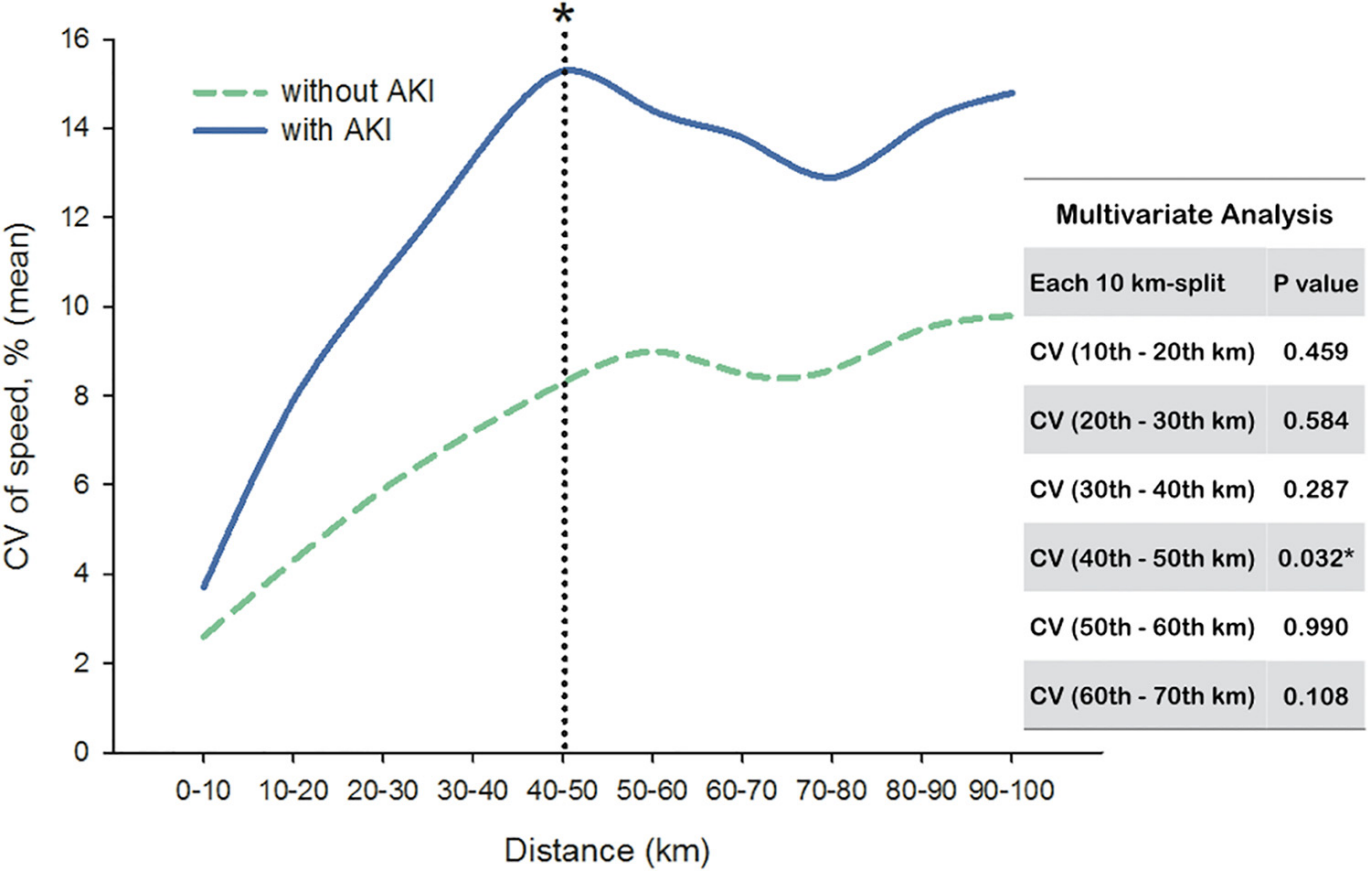
Coefficient of variation (CV) of speed in each 10 km-split between runners with or without AKI (Stage II) development. Only CV in 40^th^– 50^th^ km-split showed significant difference (*p* = 0.032) between each group in multivariate analysis*.

On the contrary, runners with or without AKI (Stage II) development both encountered an escalation of CV (Fig 2). From start to finish, runners who developed AKI (Stage II) experienced a steeper slope in change of CV than those without AKI. Strikingly, significant differences were found in CV between these two groups from secondary 10 km-split (10^th^– 20^th^ km-split) to 60^th^– 70^th^ km-split. For runners developing AKI (Stage II), the highest CV of speed (15.26 ± 2.90%) occurred in the 40^th^– 50^th^ km-split.

Individual CV was then applied to multiple logistic regression analysis with BMI and UA to create the predicted probability. Compared to BMI and UA, only CVs in specific 10 km-split (from 10^th^– 20^th^ km-split to 60^th^– 70^th^ km-split) were significant to predict the AKI (Stage II) development and showed discrimination ability in ROC curve (Table 4). We also used multivariate analysis to test the independent effect; however, majority of CV in different 10 km-split lost their significance associated to AKI (Stage II). Only CV in 40^th^– 50^th^ km-split showed significant difference between runners with or without AKI (Stage II) development (*p* = 0.032).

**Table 4:** Discrimination ability of CV (by ROC curve) to predict AKI (Stage II) in individualized 10 km-split.

	AUC	*P* value
CV (10^th^– 20^th^ km)	0.989	0.002
CV (20^th^– 30^th^ km)	0.932	0.007
CV (30^th^– 40^th^ km)	0.999	0.002
CV (40^th^– 50^th^ km)	0.966	0.004
CV (50^th^– 60^th^ km)	0.943	0.006
CV (60^th^– 70^th^ km)	0.909	0.011

Definition of abbreviations: AUC = Area under curve.

## Discussions

In our study, a high proportion of runners (84.6%) had laboratory evidence of AKI (Stage I or II) immediately after the race, and among them, 15.4% were in AKI Stage II. These finding was compatible to previous reports [12–14]. Although all runners with AKI had their renal function recovered on the next day, they were at risk to progress to more severe AKI if they received inadequate management during and after the high endurance exercise, such as inappropriate fluid supplement or NSAID use. In the literature, some studies examined the epidemiology of AKI in ultramarathon runners; however, there is no real-time predicting tool being used to identify runners at risk of AKI development during the ultramarathon event.

Hoffman and colleagues [29,30] reported that younger and less experienced ultramarathon runners were more at higher risk of exercise-related injuries. This finding may be dampened with years of adaptations, enhanced knowledge, and intrinsic psychological qualities. In this study, we found that runners with AKI (Stage II) were slightly older (49.50 ± 4.66 vs. 46.45 ± 9.6, *p* = 0.55) and less experienced (4.63 ± 1.49 vs. 5.67 ± 3.65, *p* = 0.59) than those without AKI (Stage II); however, there was no statistically significant difference between each group. In addition, the impact of weekly training protocol in the prior 6 months was assessed and no significant difference was identified between each group (*p* = 0.331).

Many studies [7–9] demonstrated that exertional rhabdomyolysis occurs frequently in marathon/ultramarathon runners and might be a major risk factor of AKI. This complication is characterized by skeletal muscle break-down and leakage of muscle-cell contents into blood stream, such as CK, myoglobin, and electrolytes. The probable pathogenesis results from the renal toxicity of myoglobin, which may cause tubular obstruction and toxic reaction to the kidney. In our study, serum myoglobin was significantly increased after race in all runners, but there was no significant association between pre-race myoglobin and AKI (Stage II) development (45.4 ± 6.13 vs. 49.57 ± 22.41, *p* = 0.72). This finding revealed that pre-race myoglobin level could not serve as a useful prediction factor for AKI in ultramarathon.

Dehydration is another risk factor for AKI development after endurance exercise. Although body weight loss does not mean dehydration absolutely, previous studies suggested to maintain exercise-induced body weight loss around 2–3% to prevent dehydration and improve performance [31–32]. In our study, body weight loss in first 4 hours did not show significant association to the development of AKI (Stage II) (1.75 ± 0.76 vs. 2.16 ± 1.33, *p* = 0.56). BMI was another important issue to influence hydration status. Ritz and colleagues [33] reported that higher BMI was negatively correlated with body water space and more at risk of dehydration. In our study, runners who developed AKI (Stage II) had significant higher BMI (25.23 ± 2.02 vs. 22.55 ± 1.96, *p* = 0.02) compared to those who did not developed AKI (Stage II).

It is interesting that variability of speed showed significant association to AKI (Stage II) development in this ultramarathon event. In the literature, there is little published data focused on the variability of speed in high endurance exercise, and most of them evaluated the association of performance and metabolic demand in elite runners only in shorter- and middle-distance competition [23,27]. Previous studies reported that the runner’s variability of speed has a CV range of 1 to 20% during a 3,000 m race to more than 100 km ultramarathon [23,26,27]. Although CV of speed could be influenced by factors such as altitude changes, fatigue sensation and strategic approach to speed, we can speculate that the inability to maintain running speed steadily may be attributed to physiological abnormality. In our study, runners who developed AKI (Stage II) had greater CV of speed compared to those without AKI. In 40^th^– 50^th^ km-split, CV of speed had the highest value (15.26 ± 2.90% vs. 8.27 ± 3.92%) in runners with or without AKI (Stage II), and most important, CV in this 10 km-split was the only variable associated with the AKI (Stage II) development. This finding was similar to previous reports that fatigue related to glycogen depletion could occur after 40^th^– 50^th^ km running at about 65% VO_2_ max [34,35].

This is the first study to evaluate the association between variability of running speed and AKI development. It provides a useful tool (CV of speed) to identify the AKI development early. All runners ran over a flat 400 m oval track course, which would have excluded the influence of attitude change and trail condition. Finally, we used each 10 km-split to calculate CV of speed which can minimize the effect of strategy approach to pacing.

There are some limitations in this study. First, we used lap times of each 400 m-track to calculate the running speed, not real-time GPS (Global Positioning System) velocity. Second, specific speed data were excluded manually when runners rested, micturated, or took water and food. Third, the pre-race blood samples were obtained one week before ultramarathon, not immediately before the competition because runners hesitated to receive blood drawn close to the date of competition. Fourth, the study population was limited to Asian only without including a variety of races. Finally and most importantly, the study population is relative small to get a statistically powerful analysis

## Conclusions

In conclusion, ultramarathon is a high endurance exercise associated with a variety of biochemical changes. Although the development of exercise-related AKI was not unusual in these ultra-runners, they recovered their renal function one day later. Because not all runners can routinely receive laboratory studies after race, variability of running speed (CV of speed) may offer a timely and efficient tool to identify AKI early during the competition. The importance of this proposed tool will require further validation.
