# Correction: Clinical Impact of Speed Variability to Identify Ultramarathon Runners at Risk for Acute Kidney Injury

**DOI:** 10.1371/journal.pone.0146815

**Published:** 2016-01-05

**Authors:** Sen-Kuang Hou, Yu-Hui Chiu, Yi-Fang Tsai, Ling-Chen Tai, Peter C. Hou, Chorng-Kuang How, Chen-Chang Yang, Wei-Fong Kao

An affiliation for the last author is missing. Wei-Fong Kao is also affiliated with Department of Emergency Medicine, School of Medicine, Taipei Medical University, Taipei, Taiwan.
